# A central nervous system metastasis of melanoma with stroke‐like onset of left‐lower quadrantanopsia

**DOI:** 10.1002/jgf2.301

**Published:** 2020-02-11

**Authors:** Akira Arakawa, Akihiko Mitsutake, Takuto Hideyama, Tatsuya Sato, Junko Katsumata, Tomonari Seki, Risa Maekawa, Makoto Ohno, Yoshitaka Narita, Yasushi Shiio

**Affiliations:** ^1^ Department of Neurology Tokyo Teishin Hospital Tokyo Japan; ^2^ Department of Cerebrospinal Oncology National Cancer Center Hospital Tokyo Japan

**Keywords:** central nervous system metastasis, magnetic resonance imaging, melanoma, stroke mimics, visual symptom

## Abstract

“Stroke mimics” mean diseases presenting with acute neurological impairments that are taken for stroke. Discriminating them is crucial to avoid improper treatment or delayed correct treatment. We describe a 48‐year‐old woman presenting with a sudden onset of scintillating scotoma and left‐lower quadrantanopsia. Hyperacute cerebral infarction was suspected. However, brain magnetic resonance imaging (MRI) revealed a mass at the cortico‐medullary junction in the right occipital lobe. We diagnosed her as metastatic melanoma. We suspected that neurological deficits can be attributed to seizure, and therefore introduced levetiracetam. She showed neurological improvement immediately. Our case demonstrated the importance of considering brain tumor as a differential diagnosis in patients presenting with acute‐onset neurological deficits. In addition to appropriate treatment of tumor, the use of newer antiepileptic drugs resulted in good neurological prognosis in metastatic brain tumors.

## Graphical abstract

Brain MRI and CT showed a mass lesion 2 cm in diameter at the cortico‐medullary junction in right occipital lobe. The findings were typical characteristics of metastatic melanoma.

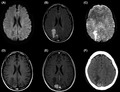

## 1 INTRODUCTION

“Stroke mimics” mean diseases presenting with a sudden onset of focal neurological impairments that are sometimes misdiagnosed as stroke. Seizure, syncope, sepsis, migraine, and brain tumor are the most common. When they come within 4.5 hours from onset, neurologists consider thrombolytic therapy, which accompanies the risk of symptomatic hemorrhage. So, discriminating them from stroke is crucial to avoid improper treatment or delayed correct treatment of the disease. We herein report a case of central nervous system (CNS) metastasis of malignant melanoma which presented with stroke‐like onset. The patient was successfully treated with levetiracetam, a newer antiepileptic drug (AED).

## 2 CASE REPORT

A 48‐year‐old woman was referred to our hospital suspected of hyperacute cerebral infarction with a chief complaint of left‐lower quadrantanopsia. She exhibited a sudden onset of scintillating scotoma one and a half hours before arrival, followed by left‐lower quadrantanopsia. On arrival, she was alert and oriented. Her vital signs were within normal limit. Neurological examination revealed left‐lower quadrantanopsia, horizontal gaze palsy, and disturbance of attention. Her National Institute of Health Stroke Scale (NIHSS) was 4. We considered undergoing thrombolytic therapy with the use of tissue plasminogen activator.

**Figure 1:**
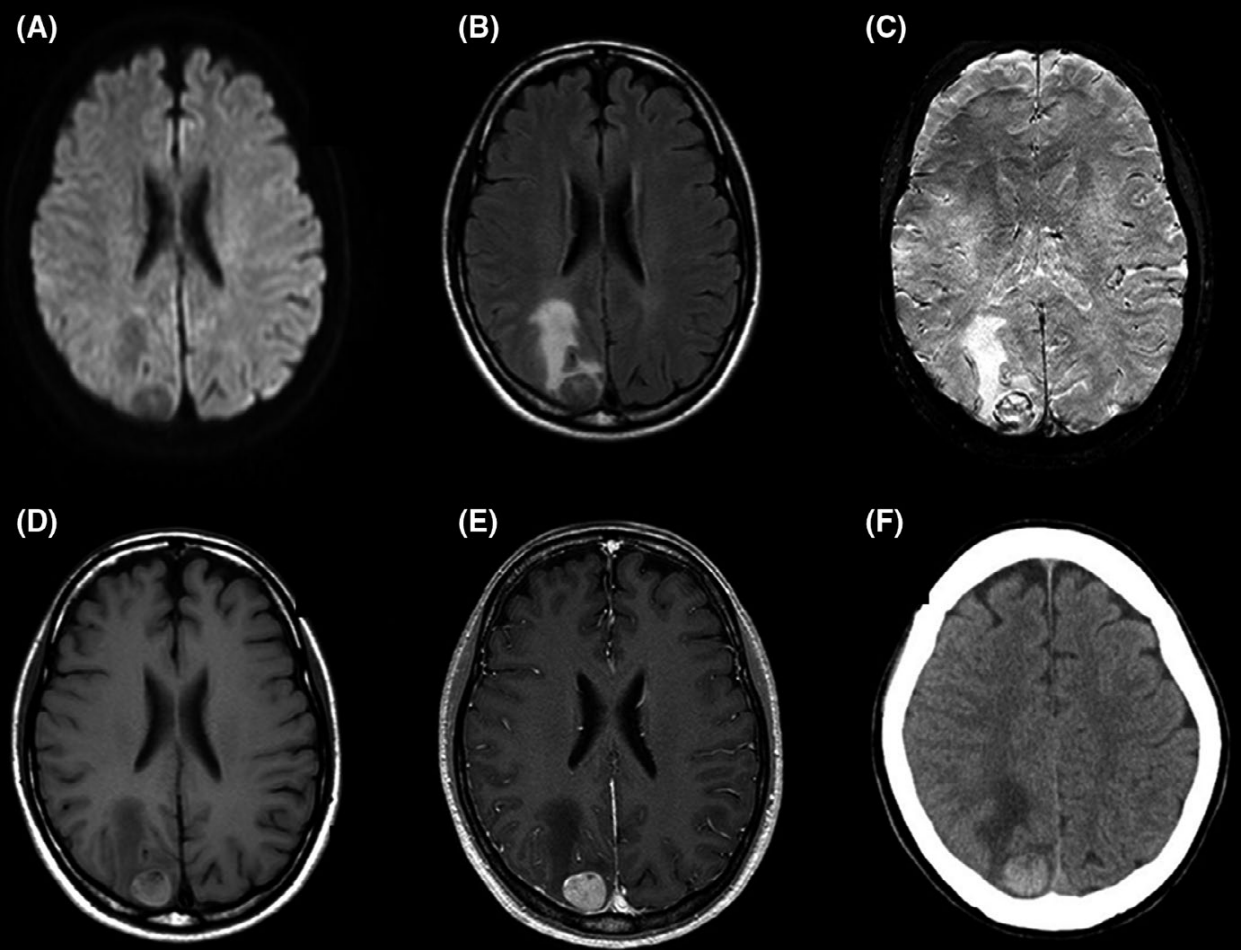
Brain magnetic resonance imaging (MRI) and computed tomography (CT) on admission. Brain MRI showed a mass lesion 2 cm in diameter at the cortico‐medullary junction in the right occipital lobe. It was hypointense on diffusion‐weighted imaging. (A) Fluid‐attenuated inversion recovery imaging revealed coexistence of hypo‐ and hyperintense areas surrounded by hyperintense lesion in white matter, suggesting edema. (B) T2*‐weighted imaging showed hypointense area at the edge. (C) The lesion was hyperintense on T1‐weighted imaging (D) and later turned out to be hyperintense on gadolinium contrast–enhanced imaging. (E) The lesion was hyperdense on CT. (F)

We performed brain MRI immediately. However, contrary to our expectation, it showed a hypointense lesion in the right occipital lobe on diffusion‐weighted images and fluid‐attenuated inversion recovery. T2*‐weighted images showed hypointense areas at the edge. This lesion was hyperintense on gadolinium contrast–enhanced T1‐weighted images. These findings were typical characteristics of metastatic melanoma (Figure 1). Signal change on T1‐weighted images excluded acute cerebral venous or sinus thrombosis, because central venous thromboses do not present signal change in this sequence in the early phase.

Her past medical history included dorsal melanoma with lung and axillary lymph node metastasis at 44 years of age. She underwent tumor and lymph node resection. She subsequently received five courses of DAV‐feron therapy with dacarbazine, nimustine, vincristine, and interferon‐beta. At the age of 46, she underwent tumor resection because of recurrence at the precordium and received interferon‐β injection therapy.

Serum laboratory examination was within normal range, including glucose (102 mg/dL) and D‐dimer (0.2 μL/mL). Cerebrospinal fluid findings revealed slightly elevated protein (53 mg/dL), but were otherwise normal. Cytology and bacterial cultures were negative. Electroencephalography (EEG) showed sharp waves in the occipital region, accompanied by reduced appearance of alpha waves. Truncal CT and abdominal ultrasonography revealed no metastatic lesion or swollen lymph node.

We made the diagnosis of melanoma CNS metastases. Stroke‐like episode was speculated to be induced by a seizure‐like mechanism from metastatic lesion. The patient was treated with levetiracetam, concentrated glycerin, fructose, carbazochrome, and tranexam immediately after admission. Quadrantanopsia and gaze palsy gradually subsided, disappearing 2 days after admission. She was discharged from our hospital 7 days after admission. Subsequently, stereotactic radiation therapy was performed at another hospital for 3 days, and follow‐up MRI after 3 months from admission showed the reduction in the size of lesion. The lesion was surgically resected 6 months after admission. For the next 1 year, the patient still followed an uneventful course without any recurrence of metastasis, seizure recurrence, or neurological deficits. One and a half years later after admission, the patient experienced recurrence of melanoma with multiple brain metastases. On brain MRI, hydrocephalus and melanomatosis cerebri were not observed. The patient eventually died 2 years after discharge from our department because of multiple‐organ failure.

## 3 DISCUSSION

Stroke mimics account for 20%–25% of suspected stroke cases in the UK^1^ and 8.8% of cases in Japan.^2^ Though the common correct diagnoses were different among reports, brain tumors account for a considerable proportion of stroke mimics (Table 1).

**Table 1:** The common correct diagnoses of stroke mimics

Causes of stroke mimics	Percentage of patients with each diagnosis		
	Okano et al^3^	Fernandes et al^2^	Gibson et al^4^
Epileptic seizure	20.4	20	19.6
Psychiatric diagnosis	15.3	9	7.4
Hypoglycemia	10.9	6	6.2
Acute aortic dissection	9.5	N/A	N/A
Drug or alcohol intoxication	9.4	2	1.6
Syncope	6.6	15	12.2
Sepsis	6.6	12	9.6
Brain tumor	5.1	7	8.2

Misdiagnosis might lead to improper treatment of the disease, especially when they came within 4.5 hours from onset, and therefore, thrombolytic therapy was considered.

With regard to brain tumor, most patients present with subacute or chronic time course. On average, symptoms developed approximately 3 weeks before the diagnosis of brain metastases (median 2 weeks).^3^ However, about 5%–10% of tumors present with a stroke‐like time course.^4^ Lewis et al reported that 11 out of 224 (5%) patients with brain tumors were initially diagnosed as “stroke.” Symptoms at onset included seizure, headache, long‐tract symptoms (focal weakness or numbness), abnormal mental state, vision change, and aphasia (Table S1). The speculated mechanisms include postseizure paralysis, focal inhibitory seizure, acute intracranial pressure changes and consequent reduced cerebral blood flow, vascular steal phenomenon, acute hemorrhage, vascular compression and resultant infarction, and tumor embolus.^5^

Seizure is a common manifestation of brain tumors. Patients with metastatic melanoma are most likely to develop seizures, with 67% of all cases.^6^ Seizure control can be achieved by AEDs in addition to antitumor treatments including surgery, radiotherapy, and chemotherapy. Classical AEDs (eg, carbamazepine, phenytoin, and sodium valproate) failed to control brain tumor–related seizures because of side effects, difficulty of maintaining therapeutic serum antiepileptic drug levels, changes in plasma albumin levels, and drug interactions.^7^ Neurological prognosis was often poor. Recently, although there are no satisfactory randomized controlled clinical trials,^8^ several prospective series suggest newer AEDs lead to lower risk of seizures and longer seizure‐free period compared to classical AEDs in patients with primary brain tumors (Table S2),^9^ and possibly metastatic brain tumors.^10^

The good neurological prognosis of the patient and the absence of recurrence could be achieved by appropriate treatment of metastatic lesion and medication with newer AEDs.

## 4 CONCLUSION

This report highlights the importance of considering brain tumor as a differential diagnosis of patients with acute‐onset neurological deficits. Non‐negligible proportion of brain tumors present with stroke‐like episodes. Newer AEDs in addition to tumor‐oriented treatments could result in good neurological prognosis.

## CONFLICT OF INTEREST

Dr Narita has received an honorarium from Chugai Pharmaceutical Co., Ltd and conducted contract research with AbbVie GK, Ono Pharmaceutical Co., Ltd., Stella Pharma Co., Ltd., Sumitomo Heavy Industries, Ltd., and Nihon Medi‐Physics Co., Ltd. The other authors state that they have no conflict of interest. All the authors state they have no conflict of interest with Otsuka Pharmaceutical Co., Ltd. or UCB Japan Co. Ltd, the distributor of levetiracetam.
